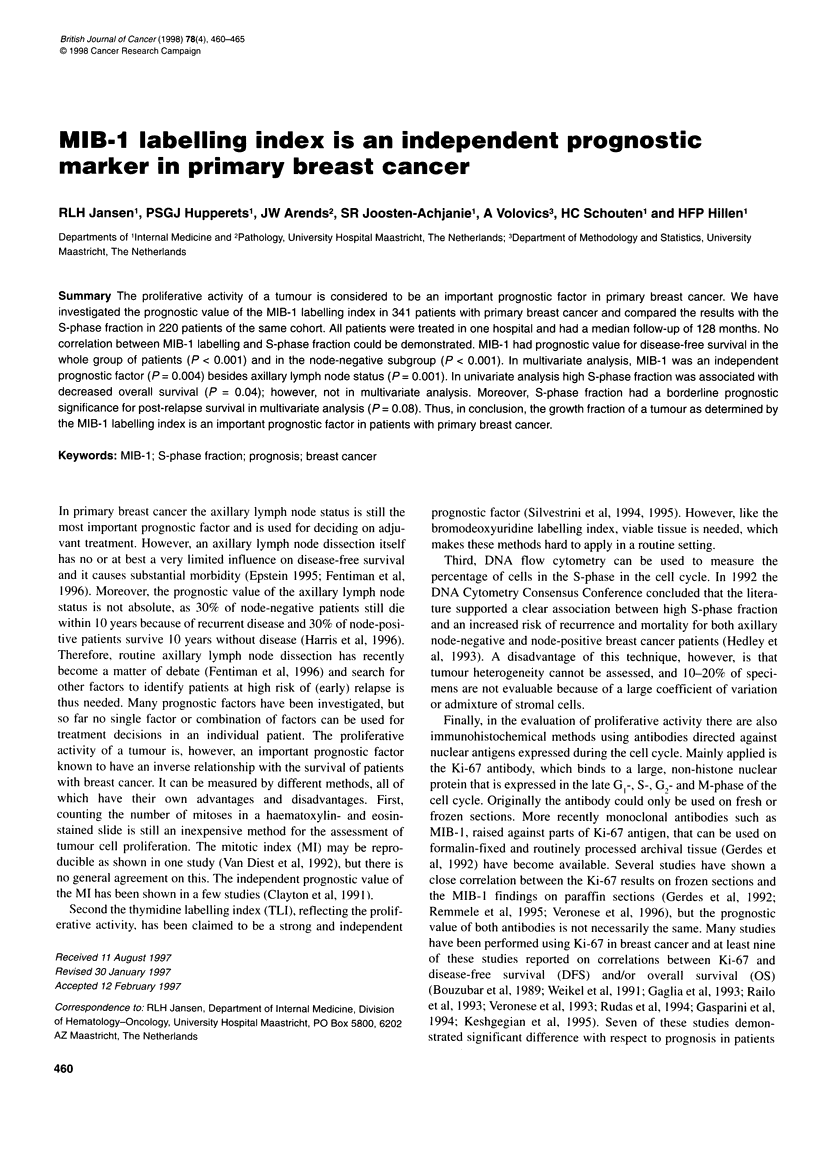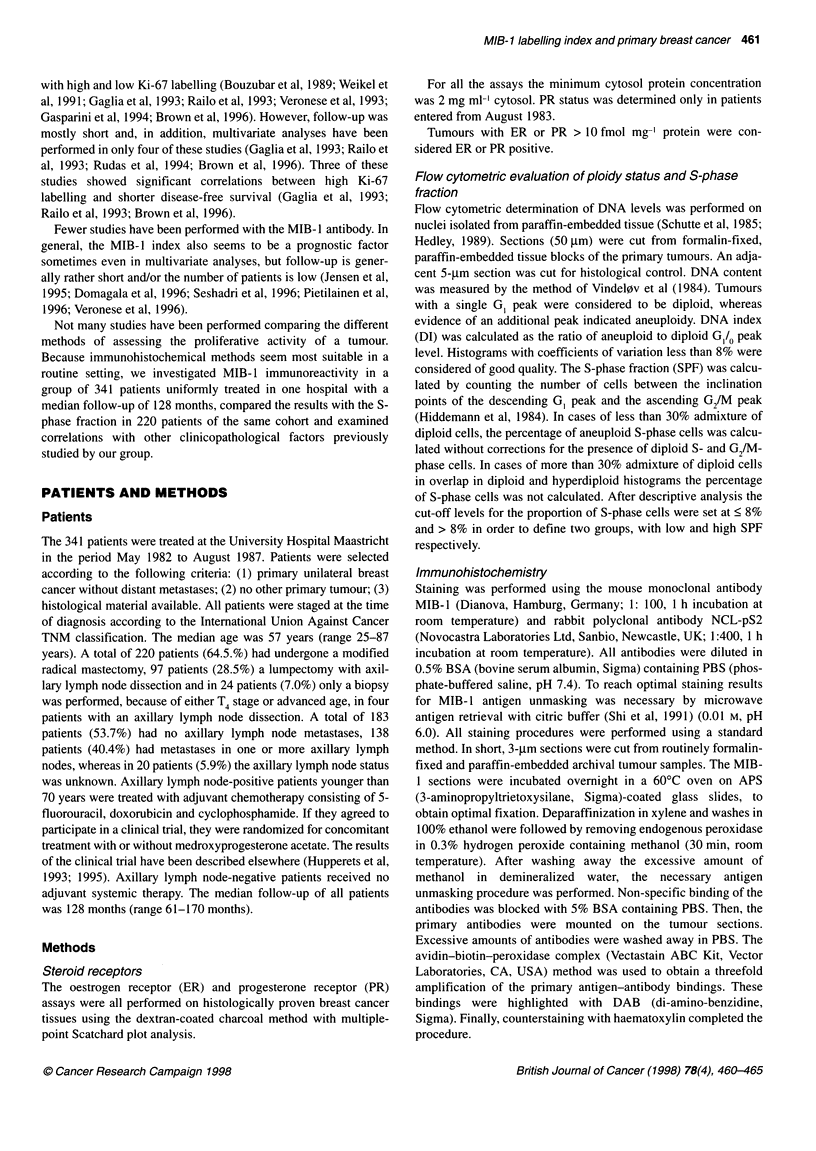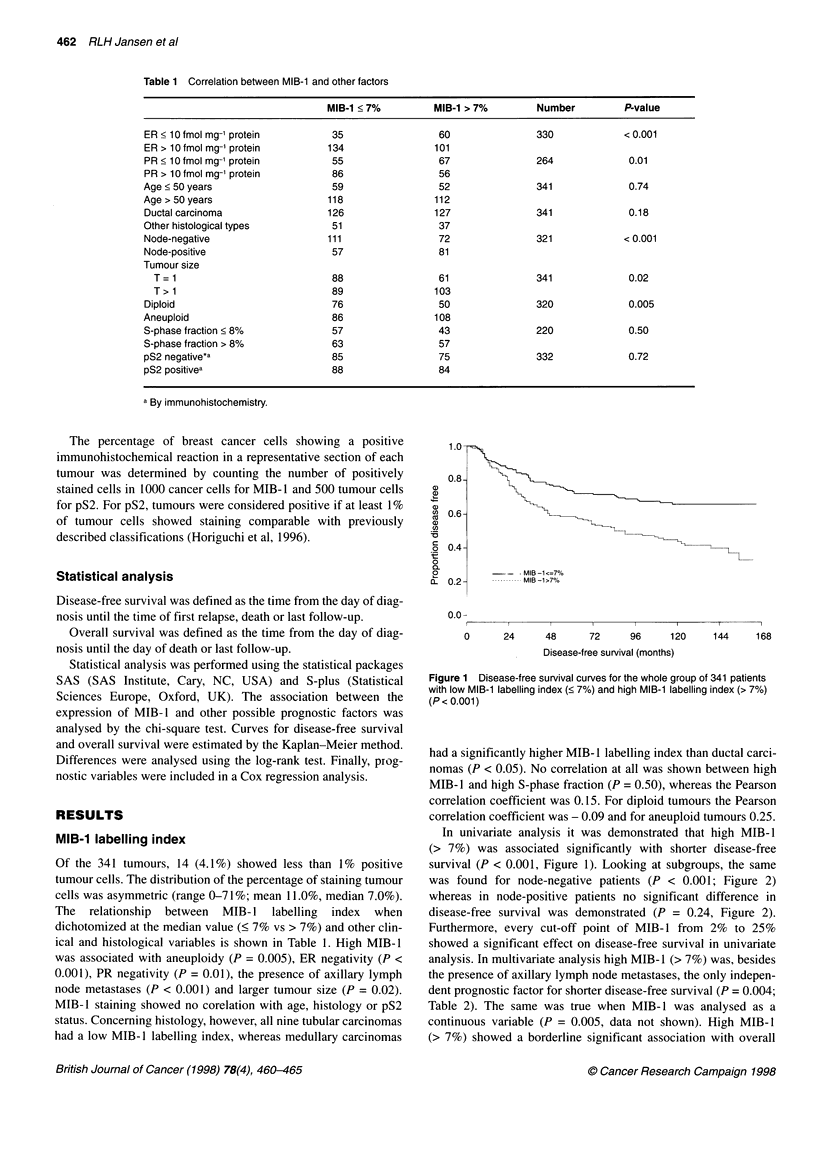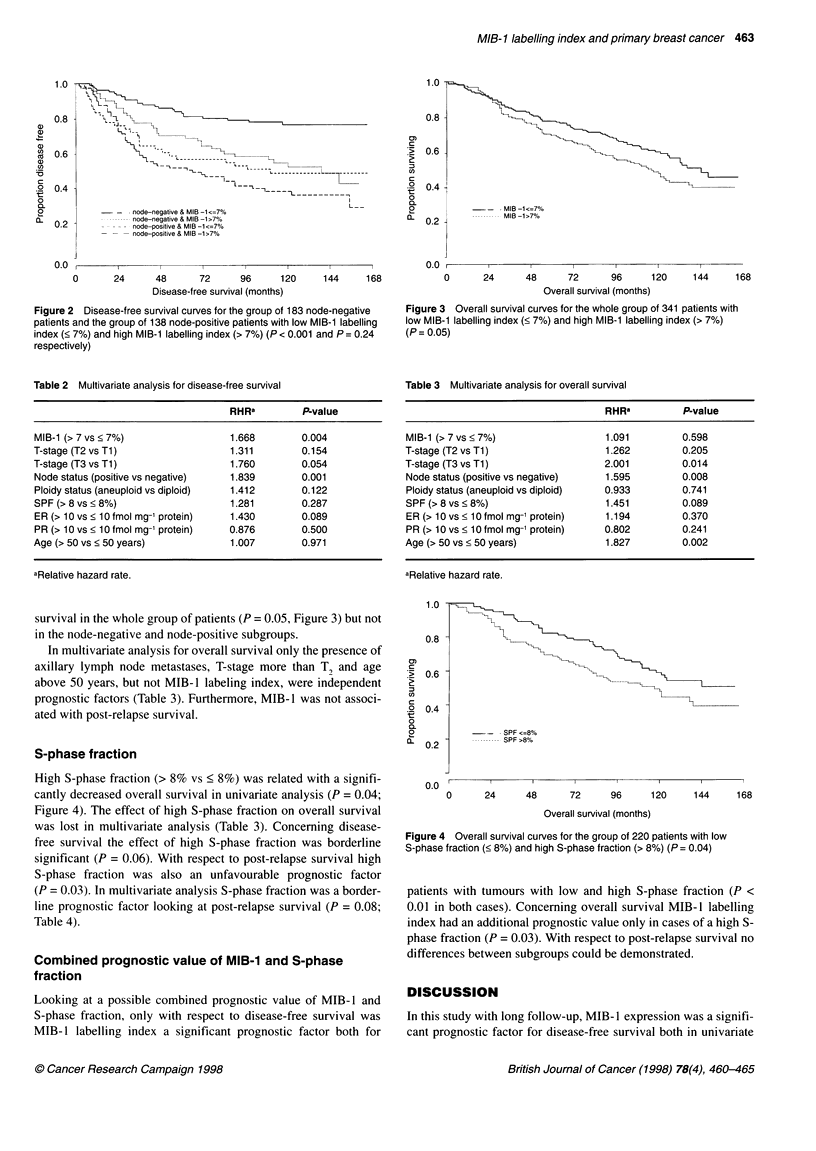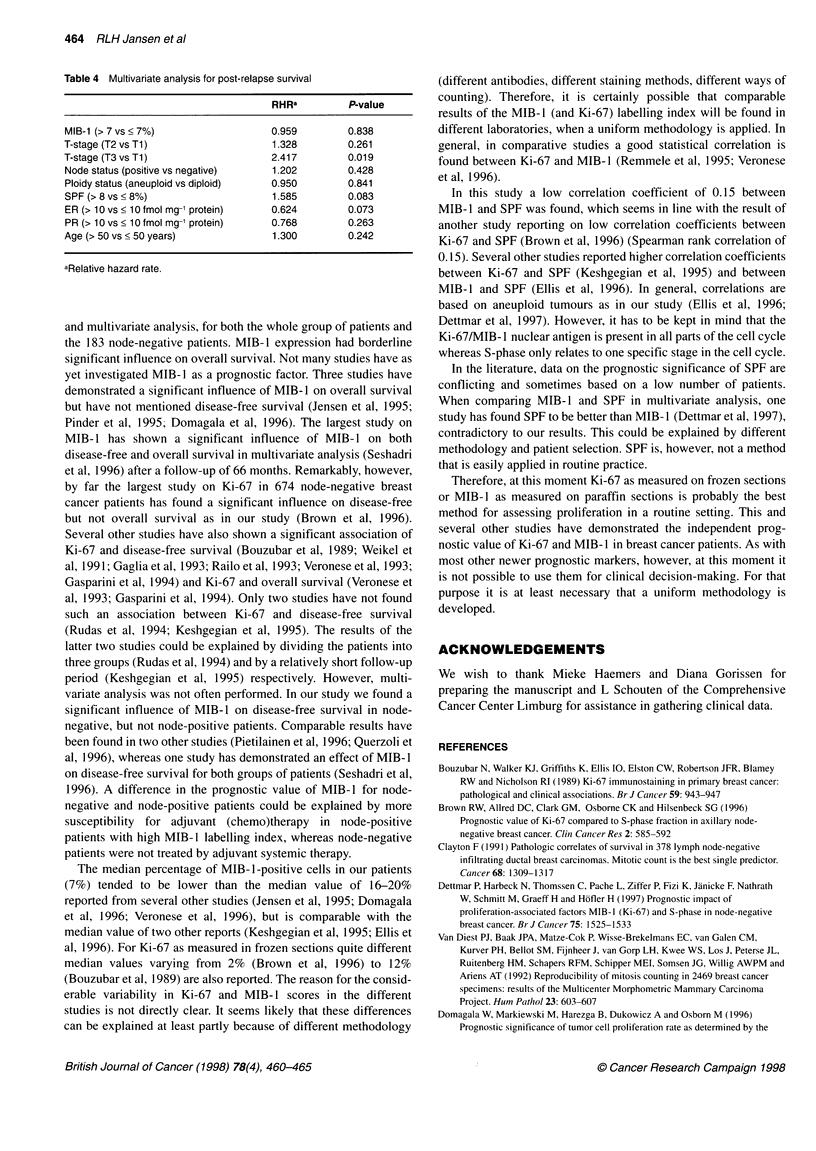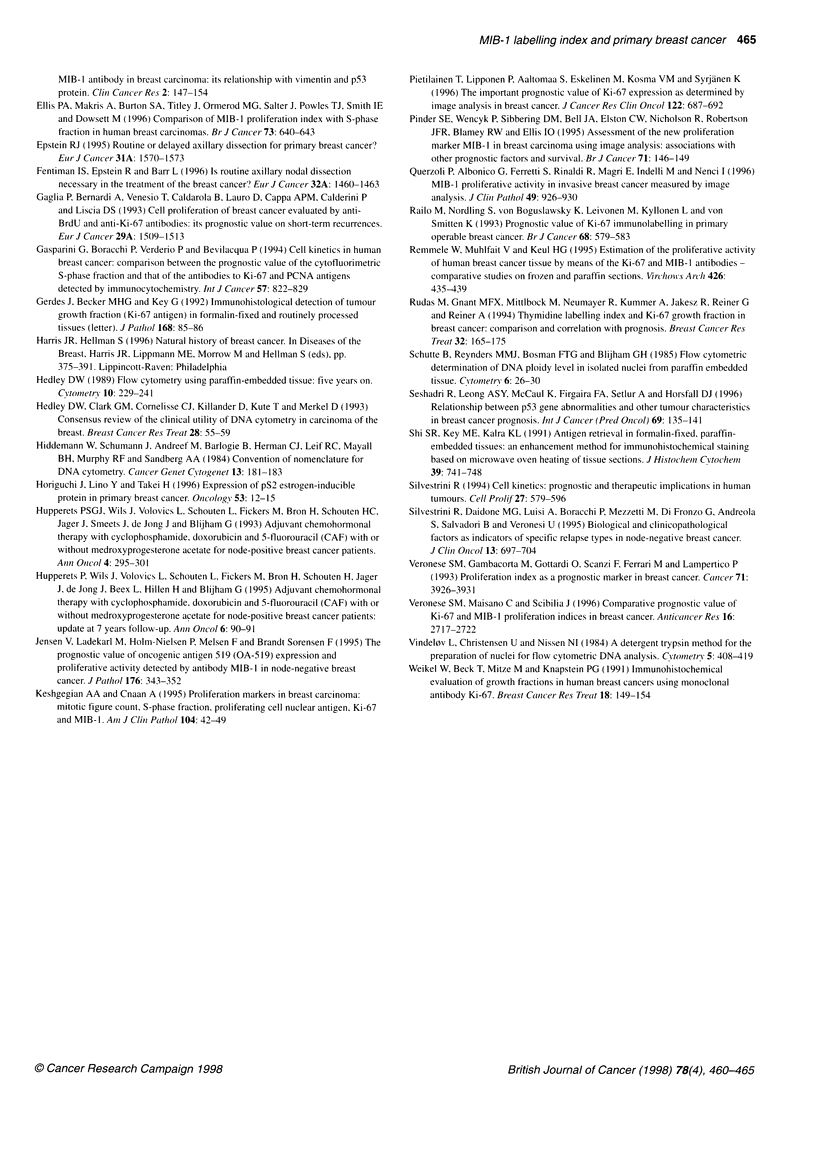# MIB-1 labelling index is an independent prognostic marker in primary breast cancer.

**DOI:** 10.1038/bjc.1998.515

**Published:** 1998-08

**Authors:** R. L. Jansen, P. S. Hupperets, J. W. Arends, S. R. Joosten-Achjanie, A. Volovics, H. C. Schouten, H. F. Hillen

**Affiliations:** Department of Internal Medicine, University Hospital Maastricht, The Netherlands.

## Abstract

The proliferative activity of a tumour is considered to be an important prognostic factor in primary breast cancer. We have investigated the prognostic value of the MIB-1 labelling index in 341 patients with primary breast cancer and compared the results with the S-phase fraction in 220 patients of the same cohort. All patients were treated in one hospital and had a median follow-up of 128 months. No correlation between MIB-1 labelling and S-phase fraction could be demonstrated. MIB-1 had prognostic value for disease-free survival in the whole group of patients (P < 0.001) and in the node-negative subgroup (P < 0.001). In multivariate analysis, MIB-1 was an independent prognostic factor (P = 0.004) besides axillary lymph node status (P = 0.001). In univariate analysis high S-phase fraction was associated with decreased overall survival (P = 0.04); however, not in multivariate analysis. Moreover, S-phase fraction had a borderline prognostic significance for post-relapse survival in multivariate analysis (P= 0.08). Thus, in conclusion, the growth fraction of a tumour as determined by the MIB-1 labelling index is an important prognostic factor in patients with primary breast cancer.


British Journal of Cancer (1998) 78(4), 460–465
© 1998 Cancer Research Campaign

### MIB-1 labelling index is an independent prognostic marker in primary breast cancer

**RLH Jansen[1], PSGJ Hupperets[1], JW Arends[2], SR Joosten-Achjanie[1], A Volovics[3], HC Schouten[1] and HFP Hillen[1]**

Departments of [1]Internal Medicine and [2]Pathology, University Hospital Maastricht, The Netherlands; [3]Department of Methodology and Statistics, University Maastricht, The Netherlands

**Summary** The proliferative activity of a tumour is considered to be an important prognostic factor in primary breast cancer. We have investigated the prognostic value of the MIB-1 labelling index in 341 patients with primary breast cancer and compared the results with the S-phase fraction in 220 patients of the same cohort. All patients were treated in one hospital and had a median follow-up of 128 months. No correlation between MIB-1 labelling and S-phase fraction could be demonstrated. MIB-1 had prognostic value for disease-free survival in the whole group of patients ($P < 0.001$) and in the node-negative subgroup ($P < 0.001$). In multivariate analysis, MIB-1 was an independent prognostic factor ($P = 0.004$) besides axillary lymph node status ($P = 0.001$). In univariate analysis high S-phase fraction was associated with decreased overall survival ($P = 0.04$); however, not in multivariate analysis. Moreover, S-phase fraction had a borderline prognostic significance for post-relapse survival in multivariate analysis ($P = 0.08$). Thus, in conclusion, the growth fraction of a tumour as determined by the MIB-1 labelling index is an important prognostic factor in patients with primary breast cancer.

**Keywords:** MIB-1; S-phase fraction; prognosis; breast cancer

In primary breast cancer the axillary lymph node status is still the most important prognostic factor and is used for deciding on adjuvant treatment. However, an axillary lymph node dissection itself has no or at best a very limited influence on disease-free survival and it causes substantial morbidity (Epstein 1995; Fentiman et al, 1996). Moreover, the prognostic value of the axillary lymph node status is not absolute, as 30% of node-negative patients still die within 10 years because of recurrent disease and 30% of node-positive patients survive 10 years without disease (Harris et al, 1996). Therefore, routine axillary lymph node dissection has recently become a matter of debate (Fentiman et al, 1996) and search for other factors to identify patients at high risk of (early) relapse is thus needed. Many prognostic factors have been investigated, but so far no single factor or combination of factors can be used for treatment decisions in an individual patient. The proliferative activity of a tumour is, however, an important prognostic factor known to have an inverse relationship with the survival of patients with breast cancer. It can be measured by different methods, all of which have their own advantages and disadvantages. First, counting the number of mitoses in a haematoxylin- and eosin-stained slide is still an inexpensive method for the assessment of tumour cell proliferation. The mitotic index (MI) may be reproducible as shown in one study (Van Diest et al, 1992), but there is no general agreement on this. The independent prognostic value of the MI has been shown in a few studies (Clayton et al, 1991).

Second the thymidine labelling index (TLI), reflecting the proliferative activity, has been claimed to be a strong and independent prognostic factor (Silvestrini et al, 1994, 1995). However, like the bromodeoxyuridine labelling index, viable tissue is needed, which makes these methods hard to apply in a routine setting.

Third, DNA flow cytometry can be used to measure the percentage of cells in the S-phase in the cell cycle. In 1992 the DNA Cytometry Consensus Conference concluded that the literature supported a clear association between high S-phase fraction and an increased risk of recurrence and mortality for both axillary node-negative and node-positive breast cancer patients (Hedley et al, 1993). A disadvantage of this technique, however, is that tumour heterogeneity cannot be assessed, and 10–20% of specimens are not evaluable because of a large coefficient of variation or admixture of stromal cells.

Finally, in the evaluation of proliferative activity there are also immunohistochemical methods using antibodies directed against nuclear antigens expressed during the cell cycle. Mainly applied is the Ki-67 antibody, which binds to a large, non-histone nuclear protein that is expressed in the late $G_1$-, S-, $G_2$- and M-phase of the cell cycle. Originally the antibody could only be used on fresh or frozen sections. More recently monoclonal antibodies such as MIB-1, raised against parts of Ki-67 antigen, that can be used on formalin-fixed and routinely processed archival tissue (Gerdes et al, 1992) have become available. Several studies have shown a close correlation between the Ki-67 results on frozen sections and the MIB-1 findings on paraffin sections (Gerdes et al, 1992; Remmele et al, 1995; Veronese et al, 1996), but the prognostic value of both antibodies is not necessarily the same. Many studies have been performed using Ki-67 in breast cancer and at least nine of these studies reported on correlations between Ki-67 and disease-free survival (DFS) and/or overall survival (OS) (Bouzubar et al, 1989; Weikel et al, 1991; Gaglia et al, 1993; Railo et al, 1993; Veronese et al, 1993; Rudas et al, 1994; Gasparini et al, 1994; Keshgegian et al, 1995). Seven of these studies demonstrated significant difference with respect to prognosis in patients

*Received 11 August 1997*
*Revised 30 January 1997*
*Accepted 12 February 1997*

*Correspondence to:* RLH Jansen, Department of Internal Medicine, Division of Hematology–Oncology, University Hospital Maastricht, PO Box 5800, 6202 AZ Maastricht, The Netherlands with high and low Ki-67 labelling (Bouzubar et al, 1989; Weikel et al, 1991; Gaglia et al, 1993; Railo et al, 1993; Veronese et al, 1993; Gasparini et al, 1994; Brown et al, 1996). However, follow-up was mostly short and, in addition, multivariate analyses have been performed in only four of these studies (Gaglia et al, 1993; Railo et al, 1993; Rudas et al, 1994; Brown et al, 1996). Three of these studies showed significant correlations between high Ki-67 labelling and shorter disease-free survival (Gaglia et al, 1993; Railo et al, 1993; Brown et al, 1996).

Fewer studies have been performed with the MIB-1 antibody. In general, the MIB-1 index also seems to be a prognostic factor sometimes even in multivariate analyses, but follow-up is generally rather short and/or the number of patients is low (Jensen et al, 1995; Domagala et al, 1996; Seshadri et al, 1996; Pietilainen et al, 1996; Veronese et al, 1996).

Not many studies have been performed comparing the different methods of assessing the proliferative activity of a tumour. Because immunohistochemical methods seem most suitable in a routine setting, we investigated MIB-1 immunoreactivity in a group of 341 patients uniformly treated in one hospital with a median follow-up of 128 months, compared the results with the S-phase fraction in 220 patients of the same cohort and examined correlations with other clinicopathological factors previously studied by our group.

### PATIENTS AND METHODS

### Patients

The 341 patients were treated at the University Hospital Maastricht in the period May 1982 to August 1987. Patients were selected according to the following criteria: (1) primary unilateral breast cancer without distant metastases; (2) no other primary tumour; (3) histological material available. All patients were staged at the time of diagnosis according to the International Union Against Cancer TNM classification. The median age was 57 years (range 25–87 years). A total of 220 patients (64.5.%) had undergone a modified radical mastectomy, 97 patients (28.5%) a lumpectomy with axillary lymph node dissection and in 24 patients (7.0%) only a biopsy was performed, because of either $T_4$ stage or advanced age, in four patients with an axillary lymph node dissection. A total of 183 patients (53.7%) had no axillary lymph node metastases, 138 patients (40.4%) had metastases in one or more axillary lymph nodes, whereas in 20 patients (5.9%) the axillary lymph node status was unknown. Axillary lymph node-positive patients younger than 70 years were treated with adjuvant chemotherapy consisting of 5-fluorouracil, doxorubicin and cyclophosphamide. If they agreed to participate in a clinical trial, they were randomized for concomitant treatment with or without medroxyprogesterone acetate. The results of the clinical trial have been described elsewhere (Hupperets et al, 1993; 1995). Axillary lymph node-negative patients received no adjuvant systemic therapy. The median follow-up of all patients was 128 months (range 61–170 months).

### Methods

*Steroid receptors*

The oestrogen receptor (ER) and progesterone receptor (PR) assays were all performed on histologically proven breast cancer tissues using the dextran-coated charcoal method with multiple-point Scatchard plot analysis.

For all the assays the minimum cytosol protein concentration was 2 mg $ml^{-1}$ cytosol. PR status was determined only in patients entered from August 1983.

Tumours with ER or PR > 10 fmol $mg^{-1}$ protein were considered ER or PR positive.

*Flow cytometric evaluation of ploidy status and S-phase fraction*

Flow cytometric determination of DNA levels was performed on nuclei isolated from paraffin-embedded tissue (Schutte et al, 1985; Hedley, 1989). Sections (50 μm) were cut from formalin-fixed, paraffin-embedded tissue blocks of the primary tumours. An adjacent 5-μm section was cut for histological control. DNA content was measured by the method of Vindeløv et al (1984). Tumours with a single $G_1$ peak were considered to be diploid, whereas evidence of an additional peak indicated aneuploidy. DNA index (DI) was calculated as the ratio of aneuploid to diploid $G_1/_0$ peak level. Histograms with coefficients of variation less than 8% were considered of good quality. The S-phase fraction (SPF) was calculated by counting the number of cells between the inclination points of the descending $G_1$ peak and the ascending $G_2$/M peak (Hiddemann et al, 1984). In cases of less than 30% admixture of diploid cells, the percentage of aneuploid S-phase cells was calculated without corrections for the presence of diploid S- and $G_2$/M-phase cells. In cases of more than 30% admixture of diploid cells in overlap in diploid and hyperdiploid histograms the percentage of S-phase cells was not calculated. After descriptive analysis the cut-off levels for the proportion of S-phase cells were set at ≤ 8% and > 8% in order to define two groups, with low and high SPF respectively.

*Immunohistochemistry*

Staining was performed using the mouse monoclonal antibody MIB-1 (Dianova, Hamburg, Germany; 1: 100, 1 h incubation at room temperature) and rabbit polyclonal antibody NCL-pS2 (Novocastra Laboratories Ltd, Sanbio, Newcastle, UK; 1:400, 1 h incubation at room temperature). All antibodies were diluted in 0.5% BSA (bovine serum albumin, Sigma) containing PBS (phosphate-buffered saline, pH 7.4). To reach optimal staining results for MIB-1 antigen unmasking was necessary by microwave antigen retrieval with citric buffer (Shi et al, 1991) (0.01 M, pH 6.0). All staining procedures were performed using a standard method. In short, 3-μm sections were cut from routinely formalin-fixed and paraffin-embedded archival tumour samples. The MIB-1 sections were incubated overnight in a 60°C oven on APS (3-aminopropyltrietoxysilane, Sigma)-coated glass slides, to obtain optimal fixation. Deparaffinization in xylene and washes in 100% ethanol were followed by removing endogenous peroxidase in 0.3% hydrogen peroxide containing methanol (30 min, room temperature). After washing away the excessive amount of methanol in demineralized water, the necessary antigen unmasking procedure was performed. Non-specific binding of the antibodies was blocked with 5% BSA containing PBS. Then, the primary antibodies were mounted on the tumour sections. Excessive amounts of antibodies were washed away in PBS. The avidin–biotin–peroxidase complex (Vectastain ABC Kit, Vector Laboratories, CA, USA) method was used to obtain a threefold amplification of the primary antigen–antibody bindings. These bindings were highlighted with DAB (di-amino-benzidine, Sigma). Finally, counterstaining with haematoxylin completed the procedure.

© Cancer Research Campaign 1998

**Table 1** Correlation between MIB-1 and other factors

| | MIB-1 ≤ 7% | MIB-1 > 7% | Number | *P*-value |
|---|---|---|---|---|
| ER ≤ 10 fmol mg$^{-1}$ protein | 35 | 60 | 330 | < 0.001 |
| ER > 10 fmol mg$^{-1}$ protein | 134 | 101 | | |
| PR ≤ 10 fmol mg$^{-1}$ protein | 55 | 67 | 264 | 0.01 |
| PR > 10 fmol mg$^{-1}$ protein | 86 | 56 | | |
| Age ≤ 50 years | 59 | 52 | 341 | 0.74 |
| Age > 50 years | 118 | 112 | | |
| Ductal carcinoma | 126 | 127 | 341 | 0.18 |
| Other histological types | 51 | 37 | | |
| Node-negative | 111 | 72 | 321 | < 0.001 |
| Node-positive | 57 | 81 | | |
| Tumour size | | | | |
| T = 1 | 88 | 61 | 341 | 0.02 |
| T > 1 | 89 | 103 | | |
| Diploid | 76 | 50 | 320 | 0.005 |
| Aneuploid | 86 | 108 | | |
| S-phase fraction ≤ 8% | 57 | 43 | 220 | 0.50 |
| S-phase fraction > 8% | 63 | 57 | | |
| pS2 negative*[a] | 85 | 75 | 332 | 0.72 |
| pS2 positive[a] | 88 | 84 | | |

[a] By immunohistochemistry.

The percentage of breast cancer cells showing a positive immunohistochemical reaction in a representative section of each tumour was determined by counting the number of positively stained cells in 1000 cancer cells for MIB-1 and 500 tumour cells for pS2. For pS2, tumours were considered positive if at least 1% of tumour cells showed staining comparable with previously described classifications (Horiguchi et al, 1996).

### Statistical analysis

Disease-free survival was defined as the time from the day of diagnosis until the time of first relapse, death or last follow-up.

Overall survival was defined as the time from the day of diagnosis until the day of death or last follow-up.

Statistical analysis was performed using the statistical packages SAS (SAS Institute, Cary, NC, USA) and S-plus (Statistical Sciences Europe, Oxford, UK). The association between the expression of MIB-1 and other possible prognostic factors was analysed by the chi-square test. Curves for disease-free survival and overall survival were estimated by the Kaplan–Meier method. Differences were analysed using the log-rank test. Finally, prognostic variables were included in a Cox regression analysis.

### RESULTS

### MIB-1 labelling index

Of the 341 tumours, 14 (4.1%) showed less than 1% positive tumour cells. The distribution of the percentage of staining tumour cells was asymmetric (range 0–71%; mean 11.0%, median 7.0%). The relationship between MIB-1 labelling index when dichotomized at the median value (≤ 7% vs > 7%) and other clinical and histological variables is shown in Table 1. High MIB-1 was associated with aneuploidy ($P$ = 0.005), ER negativity ($P$ < 0.001), PR negativity ($P$ = 0.01), the presence of axillary lymph node metastases ($P$ < 0.001) and larger tumour size ($P$ = 0.02). MIB-1 staining showed no corelation with age, histology or pS2 status. Concerning histology, however, all nine tubular carcinomas had a low MIB-1 labelling index, whereas medullary carcinomas

**Figure 1** Disease-free survival curves for the whole group of 341 patients with low MIB-1 labelling index (≤ 7%) and high MIB-1 labelling index (> 7%) ($P$ < 0.001)

had a significantly higher MIB-1 labelling index than ductal carcinomas ($P$ < 0.05). No correlation at all was shown between high MIB-1 and high S-phase fraction ($P$ = 0.50), whereas the Pearson correlation coefficient was 0.15. For diploid tumours the Pearson correlation coefficient was – 0.09 and for aneuploid tumours 0.25.

In univariate analysis it was demonstrated that high MIB-1 (> 7%) was associated significantly with shorter disease-free survival ($P$ < 0.001, Figure 1). Looking at subgroups, the same was found for node-negative patients ($P$ < 0.001; Figure 2) whereas in node-positive patients no significant difference in disease-free survival was demonstrated ($P$ = 0.24, Figure 2). Furthermore, every cut-off point of MIB-1 from 2% to 25% showed a significant effect on disease-free survival in univariate analysis. In multivariate analysis high MIB-1 (> 7%) was, besides the presence of axillary lymph node metastases, the only independent prognostic factor for shorter disease-free survival ($P$ = 0.004; Table 2). The same was true when MIB-1 was analysed as a continuous variable ($P$ = 0.005, data not shown). High MIB-1 (> 7%) showed a borderline significant association with overall

© Cancer Research Campaign 1998

**Figure 2** Disease-free survival curves for the group of 183 node-negative patients and the group of 138 node-positive patients with low MIB-1 labelling index (≤ 7%) and high MIB-1 labelling index (> 7%) ($P < 0.001$ and $P = 0.24$ respectively)

**Figure 3** Overall survival curves for the whole group of 341 patients with low MIB-1 labelling index (≤ 7%) and high MIB-1 labelling index (> 7%) ($P = 0.05$)

**Table 2** Multivariate analysis for disease-free survival

| | RHR[a] | *P*-value |
|---|---|---|
| MIB-1 (> 7 vs ≤ 7%) | 1.668 | 0.004 |
| T-stage (T2 vs T1) | 1.311 | 0.154 |
| T-stage (T3 vs T1) | 1.760 | 0.054 |
| Node status (positive vs negative) | 1.839 | 0.001 |
| Ploidy status (aneuploid vs diploid) | 1.412 | 0.122 |
| SPF (> 8 vs ≤ 8%) | 1.281 | 0.287 |
| ER (> 10 vs ≤ 10 fmol mg$^{-1}$ protein) | 1.430 | 0.089 |
| PR (> 10 vs ≤ 10 fmol mg$^{-1}$ protein) | 0.876 | 0.500 |
| Age (> 50 vs ≤ 50 years) | 1.007 | 0.971 |

[a]Relative hazard rate.

**Table 3** Multivariate analysis for overall survival

| | RHR[a] | *P*-value |
|---|---|---|
| MIB-1 (> 7 vs ≤ 7%) | 1.091 | 0.598 |
| T-stage (T2 vs T1) | 1.262 | 0.205 |
| T-stage (T3 vs T1) | 2.001 | 0.014 |
| Node status (positive vs negative) | 1.595 | 0.008 |
| Ploidy status (aneuploid vs diploid) | 0.933 | 0.741 |
| SPF (> 8 vs ≤ 8%) | 1.451 | 0.089 |
| ER (> 10 vs ≤ 10 fmol mg$^{-1}$ protein) | 1.194 | 0.370 |
| PR (> 10 vs ≤ 10 fmol mg$^{-1}$ protein) | 0.802 | 0.241 |
| Age (> 50 vs ≤ 50 years) | 1.827 | 0.002 |

[a]Relative hazard rate.

survival in the whole group of patients ($P = 0.05$, Figure 3) but not in the node-negative and node-positive subgroups.

In multivariate analysis for overall survival only the presence of axillary lymph node metastases, T-stage more than $T_2$ and age above 50 years, but not MIB-1 labeling index, were independent prognostic factors (Table 3). Furthermore, MIB-1 was not associated with post-relapse survival.

### S-phase fraction

High S-phase fraction (> 8% vs ≤ 8%) was related with a significantly decreased overall survival in univariate analysis ($P = 0.04$; Figure 4). The effect of high S-phase fraction on overall survival was lost in multivariate analysis (Table 3). Concerning disease-free survival the effect of high S-phase fraction was borderline significant ($P = 0.06$). With respect to post-relapse survival high S-phase fraction was also an unfavourable prognostic factor ($P = 0.03$). In multivariate analysis S-phase fraction was a borderline prognostic factor looking at post-relapse survival ($P = 0.08$; Table 4).

### Combined prognostic value of MIB-1 and S-phase fraction

Looking at a possible combined prognostic value of MIB-1 and S-phase fraction, only with respect to disease-free survival was MIB-1 labelling index a significant prognostic factor both for

**Figure 4** Overall survival curves for the group of 220 patients with low S-phase fraction (≤ 8%) and high S-phase fraction (> 8%) ($P = 0.04$)

patients with tumours with low and high S-phase fraction ($P < 0.01$ in both cases). Concerning overall survival MIB-1 labelling index had an additional prognostic value only in cases of a high S-phase fraction ($P = 0.03$). With respect to post-relapse survival no differences between subgroups could be demonstrated.

### DISCUSSION

In this study with long follow-up, MIB-1 expression was a significant prognostic factor for disease-free survival both in univariate

© Cancer Research Campaign 1998

**Table 4** Multivariate analysis for post-relapse survival

| | RHR[a] | P-value |
|---|---|---|
| MIB-1 (> 7 vs ≤ 7%) | 0.959 | 0.838 |
| T-stage (T2 vs T1) | 1.328 | 0.261 |
| T-stage (T3 vs T1) | 2.417 | 0.019 |
| Node status (positive vs negative) | 1.202 | 0.428 |
| Ploidy status (aneuploid vs diploid) | 0.950 | 0.841 |
| SPF (> 8 vs ≤ 8%) | 1.585 | 0.083 |
| ER (> 10 vs ≤ 10 fmol $mg^{-1}$ protein) | 0.624 | 0.073 |
| PR (> 10 vs ≤ 10 fmol $mg^{-1}$ protein) | 0.768 | 0.263 |
| Age (> 50 vs ≤ 50 years) | 1.300 | 0.242 |

[a]Relative hazard rate.

and multivariate analysis, for both the whole group of patients and the 183 node-negative patients. MIB-1 expression had borderline significant influence on overall survival. Not many studies have as yet investigated MIB-1 as a prognostic factor. Three studies have demonstrated a significant influence of MIB-1 on overall survival but have not mentioned disease-free survival (Jensen et al, 1995; Pinder et al, 1995; Domagala et al, 1996). The largest study on MIB-1 has shown a significant influence of MIB-1 on both disease-free and overall survival in multivariate analysis (Seshadri et al, 1996) after a follow-up of 66 months. Remarkably, however, by far the largest study on Ki-67 in 674 node-negative breast cancer patients has found a significant influence on disease-free but not overall survival as in our study (Brown et al, 1996). Several other studies have also shown a significant association of Ki-67 and disease-free survival (Bouzubar et al, 1989; Weikel et al, 1991; Gaglia et al, 1993; Railo et al, 1993; Veronese et al, 1993; Gasparini et al, 1994) and Ki-67 and overall survival (Veronese et al, 1993; Gasparini et al, 1994). Only two studies have not found such an association between Ki-67 and disease-free survival (Rudas et al, 1994; Keshgegian et al, 1995). The results of the latter two studies could be explained by dividing the patients into three groups (Rudas et al, 1994) and by a relatively short follow-up period (Keshgegian et al, 1995) respectively. However, multivariate analysis was not often performed. In our study we found a significant influence of MIB-1 on disease-free survival in node-negative, but not node-positive patients. Comparable results have been found in two other studies (Pietilainen et al, 1996; Querzoli et al, 1996), whereas one study has demonstrated an effect of MIB-1 on disease-free survival for both groups of patients (Seshadri et al, 1996). A difference in the prognostic value of MIB-1 for node-negative and node-positive patients could be explained by more susceptibility for adjuvant (chemo)therapy in node-positive patients with high MIB-1 labelling index, whereas node-negative patients were not treated by adjuvant systemic therapy.

The median percentage of MIB-1-positive cells in our patients (7%) tended to be lower than the median value of 16–20% reported from several other studies (Jensen et al, 1995; Domagala et al, 1996; Veronese et al, 1996), but is comparable with the median value of two other reports (Keshgegian et al, 1995; Ellis et al, 1996). For Ki-67 as measured in frozen sections quite different median values varying from 2% (Brown et al, 1996) to 12% (Bouzubar et al, 1989) are also reported. The reason for the considerable variability in Ki-67 and MIB-1 scores in the different studies is not directly clear. It seems likely that these differences can be explained at least partly because of different methodology (different antibodies, different staining methods, different ways of counting). Therefore, it is certainly possible that comparable results of the MIB-1 (and Ki-67) labelling index will be found in different laboratories, when a uniform methodology is applied. In general, in comparative studies a good statistical correlation is found between Ki-67 and MIB-1 (Remmele et al, 1995; Veronese et al, 1996).

In this study a low correlation coefficient of 0.15 between MIB-1 and SPF was found, which seems in line with the result of another study reporting on low correlation coefficients between Ki-67 and SPF (Brown et al, 1996) (Spearman rank correlation of 0.15). Several other studies reported higher correlation coefficients between Ki-67 and SPF (Keshgegian et al, 1995) and between MIB-1 and SPF (Ellis et al, 1996). In general, correlations are based on aneuploid tumours as in our study (Ellis et al, 1996; Dettmar et al, 1997). However, it has to be kept in mind that the Ki-67/MIB-1 nuclear antigen is present in all parts of the cell cycle whereas S-phase only relates to one specific stage in the cell cycle.

In the literature, data on the prognostic significance of SPF are conflicting and sometimes based on a low number of patients. When comparing MIB-1 and SPF in multivariate analysis, one study has found SPF to be better than MIB-1 (Dettmar et al, 1997), contradictory to our results. This could be explained by different methodology and patient selection. SPF is, however, not a method that is easily applied in routine practice.

Therefore, at this moment Ki-67 as measured on frozen sections or MIB-1 as measured on paraffin sections is probably the best method for assessing proliferation in a routine setting. This and several other studies have demonstrated the independent prognostic value of Ki-67 and MIB-1 in breast cancer patients. As with most other newer prognostic markers, however, at this moment it is not possible to use them for clinical decision-making. For that purpose it is at least necessary that a uniform methodology is developed.

### ACKNOWLEDGEMENTS

We wish to thank Mieke Haemers and Diana Gorissen for preparing the manuscript and L Schouten of the Comprehensive Cancer Center Limburg for assistance in gathering clinical data.

### REFERENCES

Bouzubar N, Walker KJ, Griffiths K, Ellis IO, Elston CW, Robertson JFR, Blamey RW and Nicholson RI (1989) Ki-67 immunostaining in primary breast cancer: pathological and clinical associations. *Br J Cancer* **59**: 943–947

Brown RW, Allred DC, Clark GM, Osborne CK and Hilsenbeck SG (1996) Prognostic value of Ki-67 compared to S-phase fraction in axillary node-negative breast cancer. *Clin Cancer Res* **2**: 585–592

Clayton F (1991) Pathologic correlates of survival in 378 lymph node-negative infiltrating ductal breast carcinomas. Mitotic count is the best single predictor. *Cancer* **68**: 1309–1317

Dettmar P, Harbeck N, Thomssen C, Pache L, Ziffer P, Fizi K, Jänicke F, Nathrath W, Schmitt M, Graeff H and Höfler H (1997) Prognostic impact of proliferation-associated factors MIB-1 (Ki-67) and S-phase in node-negative breast cancer. *Br J Cancer* **75**: 1525–1533

Van Diest PJ, Baak JPA, Matze-Cok P, Wisse-Brekelmans EC, van Galen CM, Kurver PH, Bellot SM, Fijnheer J, van Gorp LH, Kwee WS, Los J, Peterse JL, Ruitenberg HM, Schapers RFM, Schipper MEI, Somsen JG, Willig AWPM and Ariens AT (1992) Reproducibility of mitosis counting in 2469 breast cancer specimens: results of the Multicenter Morphometric Mammary Carcinoma Project. *Hum Pathol* **23**: 603–607

Domagala W, Markiewski M, Harezga B, Dukowicz A and Osborn M (1996) Prognostic significance of tumor cell proliferation rate as determined by the

© Cancer Research Campaign 1998

MIB-1 antibody in breast carcinoma: its relationship with vimentin and p53 protein. *Clin Cancer Res* **2**: 147–154

Ellis PA, Makris A, Burton SA, Titley J, Ormerod MG, Salter J, Powles TJ, Smith IE and Dowsett M (1996) Comparison of MIB-1 proliferation index with S-phase fraction in human breast carcinomas. *Br J Cancer* **73**: 640–643

Epstein RJ (1995) Routine or delayed axillary dissection for primary breast cancer? *Eur J Cancer* **31A**: 1570–1573

Fentiman IS, Epstein R and Barr L (1996) Is routine axillary nodal dissection necessary in the treatment of the breast cancer? *Eur J Cancer* **32A**: 1460–1463

Gaglia P, Bernardi A, Venesio T, Caldarola B, Lauro D, Cappa APM, Calderini P and Liscia DS (1993) Cell proliferation of breast cancer evaluated by anti-BrdU and anti-Ki-67 antibodies: its prognostic value on short-term recurrences. *Eur J Cancer* **29A**: 1509–1513

Gasparini G, Boracchi P, Verderio P and Bevilacqua P (1994) Cell kinetics in human breast cancer: comparison between the prognostic value of the cytofluorimetric S-phase fraction and that of the antibodies to Ki-67 and PCNA antigens detected by immunocytochemistry. *Int J Cancer* **57**: 822–829

Gerdes J, Becker MHG and Key G (1992) Immunohistological detection of tumour growth fraction (Ki-67 antigen) in formalin-fixed and routinely processed tissues (letter). *J Pathol* **168**: 85–86

Harris JR, Hellman S (1996) Natural history of breast cancer. In Diseases of the Breast, Harris JR, Lippmann ME, Morrow M and Hellman S (eds), pp. 375–391. Lippincott-Raven: Philadelphia

Hedley DW (1989) Flow cytometry using paraffin-embedded tissue: five years on. *Cytometry* **10**: 229–241

Hedley DW, Clark GM, Cornelisse CJ, Killander D, Kute T and Merkel D (1993) Consensus review of the clinical utility of DNA cytometry in carcinoma of the breast. *Breast Cancer Res Treat* **28**: 55–59

Hiddemann W, Schumann J, Andreef M, Barlogie B, Herman CJ, Leif RC, Mayall BH, Murphy RF and Sandberg AA (1984) Convention of nomenclature for DNA cytometry. *Cancer Genet Cytogenet* **13**: 181–183

Horiguchi J, Lino Y and Takei H (1996) Expression of pS2 estrogen-inducible protein in primary breast cancer. *Oncology* **53**: 12–15

Hupperets PSGJ, Wils J, Volovics L, Schouten L, Fickers M, Bron H, Schouten HC, Jager J, Smeets J, de Jong J and Blijham G (1993) Adjuvant chemohormonal therapy with cyclophosphamide, doxorubicin and 5-fluorouracil (CAF) with or without medroxyprogesterone acetate for node-positive breast cancer patients. *Ann Oncol* **4**: 295–301

Hupperets P, Wils J, Volovics L, Schouten L, Fickers M, Bron H, Schouten H, Jager J, de Jong J, Beex L, Hillen H and Blijham G (1995) Adjuvant chemohormonal therapy with cyclophosphamide, doxorubicin and 5-fluorouracil (CAF) with or without medroxyprogesterone acetate for node-positive breast cancer patients: update at 7 years follow-up. *Ann Oncol* **6**: 90–91

Jensen V, Ladekarl M, Holm-Nielsen P, Melsen F and Brandt Sorensen F (1995) The prognostic value of oncogenic antigen 519 (OA-519) expression and proliferative activity detected by antibody MIB-1 in node-negative breast cancer. *J Pathol* **176**: 343–352

Keshgegian AA and Cnaan A (1995) Proliferation markers in breast carcinoma: mitotic figure count, S-phase fraction, proliferating cell nuclear antigen, Ki-67 and MIB-1. *Am J Clin Pathol* **104**: 42–49

Pietilainen T, Lipponen P, Aaltomaa S, Eskelinen M, Kosma VM and Syrjänen K (1996) The important prognostic value of Ki-67 expression as determined by image analysis in breast cancer. *J Cancer Res Clin Oncol* **122**: 687–692

Pinder SE, Wencyk P, Sibbering DM, Bell JA, Elston CW, Nicholson R, Robertson JFR, Blamey RW and Ellis IO (1995) Assessment of the new proliferation marker MIB-1 in breast carcinoma using image analysis: associations with other prognostic factors and survival. *Br J Cancer* **71**: 146–149

Querzoli P, Albonico G, Ferretti S, Rinaldi R, Magri E, Indelli M and Nenci I (1996) MIB-1 proliferative activity in invasive breast cancer measured by image analysis. *J Clin Pathol* **49**: 926–930

Railo M, Nordling S, von Boguslawsky K, Leivonen M, Kyllonen L and von Smitten K (1993) Prognostic value of Ki-67 immunolabelling in primary operable breast cancer. *Br J Cancer* **68**: 579–583

Remmele W, Muhlfait V and Keul HG (1995) Estimation of the proliferative activity of human breast cancer tissue by means of the Ki-67 and MIB-1 antibodies – comparative studies on frozen and paraffin sections. *Virchows Arch* **426**: 435–439

Rudas M, Gnant MFX, Mittlbock M, Neumayer R, Kummer A, Jakesz R, Reiner G and Reiner A (1994) Thymidine labelling index and Ki-67 growth fraction in breast cancer: comparison and correlation with prognosis. *Breast Cancer Res Treat* **32**: 165–175

Schutte B, Reynders MMJ, Bosman FTG and Blijham GH (1985) Flow cytometric determination of DNA ploidy level in isolated nuclei from paraffin embedded tissue. *Cytometry* **6**: 26–30

Seshadri R, Leong ASY, McCaul K, Firgaira FA, Setlur A and Horsfall DJ (1996) Relationship between p53 gene abnormalities and other tumour characteristics in breast cancer prognosis. *Int J Cancer (Pred Oncol)* **69**: 135–141

Shi SR, Key ME, Kalra KL (1991) Antigen retrieval in formalin-fixed, paraffin-embedded tissues: an enhancement method for immunohistochemical staining based on microwave oven heating of tissue sections. *J Histochem Cytochem* **39**: 741–748

Silvestrini R (1994) Cell kinetics: prognostic and therapeutic implications in human tumours. *Cell Prolif* **27**: 579–596

Silvestrini R, Daidone MG, Luisi A, Boracchi P, Mezzetti M, Di Fronzo G, Andreola S, Salvadori B and Veronesi U (1995) Biological and clinicopathological factors as indicators of specific relapse types in node-negative breast cancer. *J Clin Oncol* **13**: 697–704

Veronese SM, Gambacorta M, Gottardi O, Scanzi F, Ferrari M and Lampertico P (1993) Proliferation index as a prognostic marker in breast cancer. *Cancer* **71**: 3926–3931

Veronese SM, Maisano C and Scibilia J (1996) Comparative prognostic value of Ki-67 and MIB-1 proliferation indices in breast cancer. *Anticancer Res* **16**: 2717–2722

Vindeløv L, Christensen U and Nissen NI (1984) A detergent trypsin method for the preparation of nuclei for flow cytometric DNA analysis. *Cytometry* **5**: 408–419

Weikel W, Beck T, Mitze M and Knapstein PG (1991) Immunohistochemical evaluation of growth fractions in human breast cancers using monoclonal antibody Ki-67. *Breast Cancer Res Treat* **18**: 149–154

© Cancer Research Campaign 1998